# MMP28 (epilysin) as a novel promoter of invasion and metastasis in gastric cancer

**DOI:** 10.1186/1471-2407-11-200

**Published:** 2011-05-26

**Authors:** Pan Jian, Tao Yanfang, Zhou Zhuan, Wang Jian, Zhu Xueming, Ni Jian

**Affiliations:** 1 Department of Hematology and Oncology, Children's Hospital of Soochow University, Suzhou, China; 2 Hillman Cancer Center Lab, Department of Pathology, Pittsburgh University, G21 5117 Center Ave. Pittsburgh, PA 15206, USA; 3 Department of Cell and Molecular Biology, Cancer Institute (Hospital), Chinese Academy of Medical Sciences, Peking Union Medical College, Beijing, China; 4 Translational Research Center, Second Hospital, The Second Clinical School, Nanjing Medical University, Nanjing, China

## Abstract

****Background**:**

The purpose of this study was to investigate invasion and metastasis related genes in gastric cancer.

****Methods**:**

The transwell migration assay was used to select a highly invasive sub-line from minimally invasive parent gastric cancer cells, and gene expression was compared using a microarray. *MMP28 *upregulation was confirmed using qRT-PCR. MMP28 immunohistochemistry was performed in normal and gastric cancer specimens. Invasiveness and tumor formation of stable cells overexpressing MMP28 were tested *in vitro *and i*n vivo*.

**Results:**

*MMP28 *was overexpressed in the highly invasive sub-cell line. Immunohistochemistry revealed MMP28 expression was markedly increased in gastric carcinoma relative to normal epithelia, and was significantly associated with depth of tumor invasion, lymph node metastasis and poorer overall survival. Ectopic expression of *MMP28 *indicated MMP28 promoted tumor cell invasion *in vitro *and increased gastric carcinoma metastasis *in vivo*.

****Conclusions**:**

This study indicates MMP28 is frequently overexpressed during progression of gastric carcinoma, and contributes to tumor cell invasion and metastasis. MMP28 may be a novel therapeutic target for prevention and treatment of metastases in gastric cancer.

## Background

Gastric cancer is second only to lung cancer as the leading cause of cancer-related deaths worldwide [1]. Whereas the overall incidence of gastric cancer has declined, the incidence remains high in Asian countries [1,2]. Although the early stages of gastric cancer are curable, most patients are diagnosed with late-stage disease, which currently has limited successful therapeutic strategies [3]. Surgery and combination chemotherapies confer only modest survival benefits in advanced gastric cancer, resulting in an overall 5 year survival rate of <24% [4,5]. Therefore, understanding of the molecular and genetic factors involved in gastric cancer progression may identify novel gastric biomarkers and highlight potential avenues of investigation for targeted therapies.

Matrix metalloproteinase 28 (MMP28), also known as epilysin, is a metalloproteinase cloned originally from human keratinocytes, testis, and lung cDNA libraries [6]. In rodents, *MMP28 *is expressed in many normal adult tissues, including testis, intestine, skin, and lung, suggesting a role in tissue homeostasis [7]. Fetal expression is observed in the brain, kidney and skeletal muscle [6]. Similarly to other MMPs, *MMP28 *is overexpressed in multiple disease states [8]. In some tumors and cancer cell lines *MMP28 *expression is increased [9-11]; although in some cases MMP28 protein is downregulated in cancer compared to normal tissues [12]. In wounded skin, strong upregulation of *MMP28 *occurs in mitotic cells behind the advancing wound edge, but not in actively migrating keratinocytes which secrete other MMPs such as collagenase, stromelysin, and gelatinase [10]. Tumor necrosis factor α (TNFα), but not the ten other growth factors tested, strongly stimulated *MMP28 *expression in primary cultures of human keratinocytes [10]. A conserved region upstream of the *MMP28 *transcription initiation site contains a putative NFκB binding site. MMPs act not only as metalloproteinases, as the ability of MMPs to regulate cell behavior is becoming increasingly evident [13]. For example, overexpression of MMP28 in lung adenocarcinoma cells induces an epithelial-to-mesenchymal transition (EMT) via activation of latent TGFβ [14,15]. MMP28-induced EMT is associated with loss of E-cadherin, a major mediator of cell-cell adhesion, as well as increased expression of MMP-9 (gelatinase B) and MMP-14 (MT1-MMP). The expression of MMP28 is increased in oral squamous cell carcinoma (OSCC) compared to premalignant lesions [11]. Knockdown of *MMP28 *leads to inhibition of anchorage independent growth in both OSCC (oral squamous cell carcinomas)and esophageal carcinomas[11].

The results of this study demonstrate *MMP28 *is overexpressed in a highly invasive sub-line of PAMC82 cells. Immunohistochemical analysis revealed MMP28 is overexpressed in gastric carcinoma relative to normal epithelial cells, and MMP28 is significantly associated with depth of tumor invasion, lymph node metastasis and a poorer overall survival. Our data demonstrates *MMP28 *is frequently overexpressed during gastric carcinoma progression and contributes to tumor cell invasion and metastasis.

## Methods

### Cell lines and cell culture

Human gastric cancer cell lines PAMC82 [16], N87[17], BGC823, SNU16, SNU5, SGC7901, MGC803, AGS and MKN45 were maintained in RPMI 1640 (HyClone, Logan, UT, USA) supplemented with 10% fetal bovine serum (Invitrogen, Carlsbad, CA, USA). To select for a highly invasive subpopulation, PAMC82 cells were seeded on matrigel (BD Biosciences, Bedford, MA, USA) in 8 μm pore transwell inserts (Costar, Cambridge, MA, USA). Cells which invaded through the membrane and attached to the lower well were harvested and expanded. Serial selection of cells for increased invasiveness was continued for three generations, and the sub-lines from the three different generations were designated as PAMC82-P1, PAMC82-P2 and PAMC82-P3 respectively.

### Microarray

A 22K Human Genome Array, a product of the Human Genome Oligo Set Version 2.1 (http://www.Operon.com) was used to compare gene expression profiles in PAMC82-P3 relative to PAMC82 at the Bioassay Laboratory, CapitalBio Corporation Beijing, China. Data on the gene array is provided in supplementary data S1.

### Quantitative RT-PCR

Total cellular RNA preparation and reverse transcription of 4 μg total cellular RNA to cDNA was performed as previously described [18,19], and cDNA was diluted 1:10 and used for PCR. Using the published cDNA sequence (GenBank Accession no. AF219624) primers were designed to amplify a 258-bp product of human MMP-28 (forward: 5'-CTCATCCTCTTCAAGGGTG-3', nt 1,366 to 1,383 and reverse: 5'-GGAAGAAGATGATGGAGCCA-3', nt 1,606 to 1,623). The U6 gene was used as an endogenous control (forward 5'-GCTTCGGCAGCACATATACTAAAAT-3' and reverse 5'-CGCTTCACGAATTTGCGTGTCAT-3') amplifying a 89 bp product. Primers and probes were obtained from Applied Biosystems (Carlsbad, CA, USA) and qRT-PCR was performed as previously described [20].

### Immunohistochemical staining of gastric carcinoma tissue

MMP28 expression was determined by immunohistochemistry in 304 clinical cases of gastric cancer, of which clinical follow-up data was available for 274 patients. In addition, 30 of these specimens had paired normal gastric epithelia and another 30 had paired lymph node metastasis. Immunostaining was performed using the CSA kit (DAKO, Glostrup, Denmark) with a 1 h incubation of an anti-MMP28 antibody (M5066, 2 μg/ml, Sigma, St. Louis, MO, USA) in citrate buffer. Slides were evaluated by two pathologists and MMP28 expression was semi-quantitatively scored based on the staining intensity and percentage of cells stained. Tissues with no staining were scored as 0, faint staining, moderate or strong staining in <25% of cells scored as 1, moderate staining or strong staining in 25-50% cells scored as 2 and strong staining in >50% cells was scored 3.

### MMP28 overexpressing N87 cells

PCR primers incorporating *BamH*I (5') and *Xho*I (3') (restriction sites underlined; forward 5'-CGCGGATCCGCCGCCGCCATGGTCGCGCGCGTCGGCCTC-3' and reverse 5'-ACGTCTCGAGCCGAACAGGGCGCTCCCCGAGTTG-3') were designed to amplify and clone human *MMP28 *into the pcDNA3.1 expression vector (Invitrogen) containing a C-terminus His-6 epitope to produce the pcDNA3.1-*MMP28*-c-His vector. Sequencing of the cloned gene was performed in both directions. The pcDNA3.1-*MMP28*-c-His vector was transfected into the gastric cancer cell line N87 and stable cell lines were selected by incubation with 500 μg/ml G418 for 2 weeks.

### Western blot analysis

Proteins were separated by sodium dodecylsulphate-polyacrylamide gel (SDS-PAGE) electrophoresis, transferred to polyvinylidene difluoride membranes (PVDF; Millipore, Bedford, MA, USA), blocked and then probed with anti-MMP28 (0.2 μg/ml, M5066, Sigma, St. Louis, MO, USA) and ß-actin (1:5,000, Sigma) antibodies. After washing, the blots were incubated with horseradish peroxidase-conjugated secondary antibodies and visualized using an enhanced chemiluminescence kit (Pierce, Rockford, IL, USA).

### Matrigel chemoinvasion assay

The matrigel chemoinvasion assay was performed as previously described, with some modifications. Briefly, 4 × 10^4 ^cells were seeded onto 6.5 mm Costar transwells (Corning, Lowell, MA, USA) coated with 150 μg/ml matrigel. After incubation for 24 h at 37°C, cells which remained inside the insert were removed with a cotton swab and cells which had invaded to the lower surface of the membrane were fixed in 50:50 methanol: acetone and stained with 4,6-diamidino-2-phenylindole (DAPI). After air drying the membrane, the number of cells in three random x100 fields was counted using a fluorescence microscope. Experiments were performed in triplicate and repeated twice; therefore, the values represent the mean number of invasive cells in 18 × 100 fields of view. Differences in value distribution were analyzed using one-way ANOVA, *p *< 0.05 was considered significant.

### Xenograft assays in nude mice

Nu/nu mice were obtained from the Jackson Laboratory (Vital River, Beijing, China) and maintained in a specific pathogen-free facility at the Experimental Center of the Chinese Academy of Medical Science, which is accredited by the Chinese Association for Accreditation of Laboratory Animal Care. For the spontaneous metastasis assay, stable MMP28 over-expressing N87 cells (N87-C9 and N87-C10) or control cells (N87-Ve) were subcutaneously injected into female 4-wk-old mice. The mice were euthanized 9 weeks after injection and examined for subcutaneous tumor growth and development of metastases.

### Statistical analysis

Invasion assay results were compared using One-way ANOVA. Statistical analyses of MMP28 expression and clinicopathological data was performed using Fisher's exact test. For survival analysis, Kaplan-Meier survival curves were constructed and tested by the log-rank test, *p *< 0.05 were considered statistically significant.

## Results

### MMP28 is overexpressed in the highly invasive PAMC82-P3 sub-line

**Figure 1:**
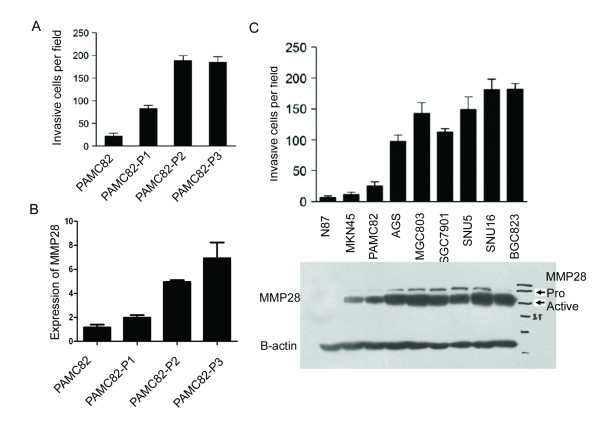
**MMP28 expression correlates with the invasive ability of gastric carcinoma cell lines**. (A) PAMC82 sub-lines with low and high invasive abilities were established and tested using the transwell invasion assay. P1-P3 cells indicated increased invasive ability, with no difference observed between P2 and P3 cells. (B) qRT-PCR analysis of *MMP28 *in PAMC82, P1 and P3 cells indicating *MMP28 *expression correlates with invasive ability. (C) MMP28 expression correlates with invasive ability in a range of gastric carcinoma cell lines. Top: transwell invasion assays of nine gastric cancer cell lines. Bottom: Western blot analysis of activated and total MMP28.

The highly invasive cancer cell line PAMC82-P3 was selected from the parental PAMC82 cell line by multiple rounds of invasion thorough matrigel. The ability of PAMC82-P3 to invade matrigel was 8-fold greater than parental PAMC82 cells, but not significantly different to the second-generation line PAMC82-P2 (Figure 1A), suggesting that invasive potential reached a plateau after two rounds of selection. The expression profiles of PAMC82 and PAMC82-P3 cells were analyzed using the 22K Human Genome Array microarray (Human Genome Oligo Set Version 2.1). 289 genes were differentially expressed by a factor of 2-fold or more, of which 213 were upregulated and 76 were downregulated in PAMC82-P3 relative to PAMC82 (Additional file 1). Most of the differentially expressed genes have been previously shown to be involved in tumor invasion and metastasis, such as *matrix metallopeptidase 1 *[21], *LOXL2 *[22], *cadherin 16 *[23], *lectin galactoside-binding soluble protein1 *[24], *thymidine phosphorylase *[25] and *LY6/PLAUR domain containing 3 *[26]. One of the differentially expressed genes, *MMP28*, was of particular interest and qRT-PCR analysis revealed *MMP28 *expression was gradually upregulated from the parental PAMC82 cells to the highly invasive PAMC82-P3 cells (Figure 1B, Additional file 2). In order to confirm this finding, MMP28 protein expression and invasive potential was examined in a range of human gastric cancer cell lines. We found that *MMP28 *expression was positively correlated with the invasive ability of the cells (Figure 1C).

### MMP28 is overexpressed in gastric carcinoma and correlates with poorer survival

**Figure 2:**
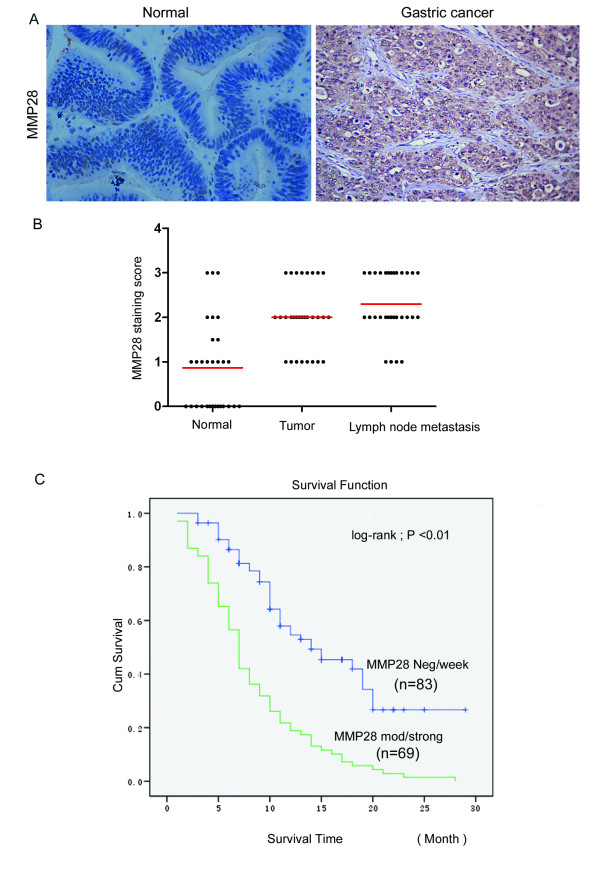
**Overexpression of MMP28 protein in gastric cancer is correlated with tumor aggressiveness and poorer prognosis**. (A) Representative images of immunohistochemical staining showing MMP28 is overexpressed in gastric cancer compared to normal tissues. (B) Semi-quantitative immunohistochemical analysis indicating MMP28 is significantly overexpressed in primary gastric carcinomas (2.08 ± 0.71) and lymph node metastases (2.36 ± 0.89) compared to normal tissues (0.94 ± 0.68, *p *< 0.01) in 30 paired samples. (C) Kaplan-Meier survival curves showing significantly reduced survival in subjects with high MMP28 expression (*p *< 0.01, log-rank test). Positive primary gastric cancer tissues were those classified as MMP28 moderate to strong (expression score of 2 and 3).

We evaluated the expression of MMP28 in 30 paired cases of gastric carcinoma tissue and normal epithelium. Compared to normal tissues, MMP28 was overexpressed in 43.3% (13/30) of the primary tumors (Figure 2A, > level 2). MMP28 was mostly localized to cytoplasm and extra cellular stroma, and this expression pattern was confirmed using fluorescent staining of N87-C9 cells transfected with MMP28 (Data not shown). MMP28 was significantly overexpressed in both primary tumors (2.08 ± 0.71) and lymph node metastatic foci (2.36 ± 0.89), compared to normal tissues (0.94 ± 0.68, Mean ± SD, semi-quantitative scores, *p *< 0.01, Figure 2B). The prognostic significance of *MMP28 *was assessed in 274 cases of gastric cancer with clinical follow-up records. Increased MMP28 expression in gastric carcinoma was correlated with depth of tumor invasion (*p *< 0.0001) and lymph node metastasis (*p *< 0.0001, Table 1). There was no significant association with MMP28 and patient age, sex or tumor differentiation. Kaplan-Meier survival analysis of 152 gastric carcinoma specimens revealed a significantly shorter overall survival times in tumors with higher MMP28 expression (*p *< 0.01, Figure 2C). Furthermore, multivariate analysis revealed that MMP28 was an independent prognostic factor in gastric cancer (Table 2).

**Table 1:** Association of MMP28 protein expression with clinico-pathological characteristics in 274 primary gastric tumors

		MMP28 expression score				
			
Variable		0	1	2	3	*P *value
**Age(yrs)**	Mean	67.0	60.8	63.7	61.0	0.374
	SD	11.0	12.8	11.9	14.3	

**Gender**	Male	29	66	70	20	0.l94
	Female	7	17	24	14	

	pT1	9	11	5	1	<0.0001*
**pT (primary tumor)**	pT2	21	49	28	8	
	pT3	11	25	50	17	
	pT4	9	8	14	8	

	pN0	28	19	6	1	<0.0001*
**pN (lymph node metastasis)**	pN1	13	43	26	4	
	pN2	9	27	45	12	
	pN3	0	4	20	17	

**pM (distant metastasis)**	pM0	49	87	94	30	0.154
	pM1	1	6	3	4	

**Table 2:** Cox multivariate analysis of MMP28 expression and clinico-pathological features in gastric cancer.

Variables	Risk ratio (95% CI)	*P *value
**pT (primary tumor)**	0.693 (0.493-0.974)	0.035*
**pN (lymph node metastasis)**	1.254 (0.978-1.608)	0.044*
**pM (distant metastasis)**	0.717 (0.349-1.470)	0.363
**Score of MMP28 expression**	1.554 (1.119-2.159)	0.008*

*Statistical analysis was performed by a χ2 test, *p *values less than 0.05 were considered to be statistically significant.

### MMP28 overexpression increases the invasive ability of gastric cancer cells

**Figure 3:**
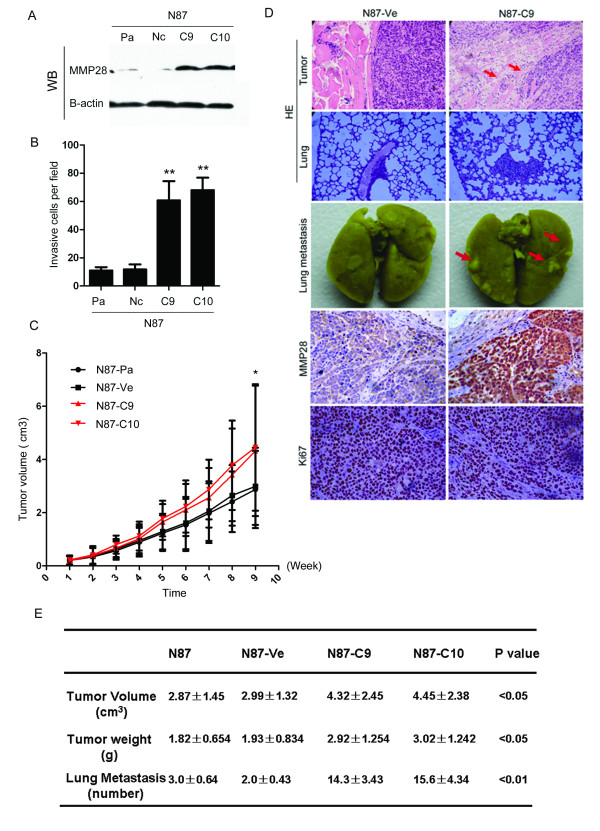
**MMP28 positively regulates tumor cell invasion and metastasis**. (A) Western Blot of MMP in parental (Pa), transfected control (N87-Ve) and MMP overexpressing (N87-C9 and N87-C10) N87 stable sub-lines. (B) Number of invasive cells in transwell invasion assays was significantly different in parental (Pa: 11.8 ± 3.19) and transfected control cells (N87-Ve: 11 ± 2.09) compared to MMP overexpressing stable N87 sub-lines (N87-C9: 60.8 ± 12.17; N87-C10: 68 ± 7.94, mean ± standard error, **p < 0.01). (B) Growth curve (n = 6) of parental (N87-Pa2: 87 ± 1.45 cm^3^), transfected control (N87-Ve: 2.99 ± 1.24 cm^3^) and MMP overexpressing N87 stable sub-lines (N87-C9: 4.32 ± 2.45 cm^3^; N87-C10: 4.45 ± 1.38 cm^3^, *p *< 0.05). (C) Top: H&E staining showing increased xenograft tumor invasion into surrounding tissue in MMP28 overexpressing cells (N87-C9) compared to control transfected cells (N87-ve), arrows indicate area of muscular invasion. Centre: lung metastasis in control transfected (N87-ve) and *MMP28 *overexpressing xenografts (N87-C9) 9 weeks after subcutaneous injection. Bottom: hematoxylin and eosin (HE) staining showing increased MMP28 expression, and no significant difference in Ki67 cell proliferation, in MMP28 overexpressing xenografts (N87-C9) compared to control transfected xenografts (N87-ve). (E) Characteristics of control and MMP overexpressing MMP28 tumor xenografts.

To examine the functional consequence of elevated MMP28 expression in gastric cancer cells, His-tagged MMP28 was overexpressed in N87 gastric cancer cells, which exhibit a low endogenous level of MMP28 (Figure 3A). In the matrigel invasion assay, invasion significantly increased in two stable MMP28 overexpressing N87 cell sub-lines (MMP28-C9: 60.8 ± 12.17; MMP28-C10: 68 ± 7.94) compared to transfected control and control cells (N87-Pa: 11 ± 2.09; N87-Nc: 11.8 ± 3.19 respectively (*p *< 0.01, Figure 3B).

### MMP28 promotes growth and spontaneous metastasis of gastric cancers in vivo

To define the function of MMP28 *in vivo*, we subcutaneously injected MMP28-overexpressing N87 clones (N87-C9 and C10) into athymic mice, and mice were euthanized 9 weeks later. MMP28 significantly promoted growth of N87 xenografts (MMP28-C9: 4.32 ± 2.45 cm^3^; MMP28-C10: 4.45 ± 1.38 cm^3^) compared to transfected control (N87-Pa: 2.87 ± 1.45 cm^3^) or control N87 cells (N87-Ve: 2.99 ± 1.24 cm^3^, *p *< 0.05 Figure 3C). Expression of MMP28 increased volume and weight of tumors, so the proliferation rate of the MMP-28 overexpressing clones C9 and C10 was analyzed, and found to be not significantly different to control cells (data not shown). Ki67 expression in all xenograft tumors groups was similar (Figure 3D). As MMP28 increased invasion and tumor volume in the absence of altered proliferation, we hypothesize MMP28 may influence expression of other genes related to tumor growth or vascular formation. MMP28 over expressing N87 (N87-MMP28) xenograft tumors showed a highly invasive pattern in HE staining sections (Figure 3D), indicating MMP28 expression significantly promotes xenograft tumor invasion into the surrounding tissue. MMP28 overexpression also significantly promoted the spontaneous metastasis of N87 cells to lung. The lungs of mice in the N87-MMP28 group had evident metastatic nodules, whereas these were barely visible on the lung surface of the control cohort (Figure 3D). H&E staining revealed a significant increase in lung metastases in MMP overexpressing N87 injected mice (MMP28-C9: 14.3 ± 3.43 and MMP28-C10: 15.6 ± 4.34) compared to mice injected with control cells (N87-Pa: 3.0 ± 0.64 and N87-Ve: 2.0 ± 0.43, *p *< 0.01 Figure 3D,E).

## Discussion

Metastasis is a multifactorial process, requiring escape of the normal microenvironment by tumor cells, entrance in and out of lymphatic or blood vessels and proliferation in distant tissue microenvironments [27]. Implicit in these stages of metastasis is the critical ability of tumor cells to invade [27]. During invasion, malignant cells reside within two major types of extracellular matrix: the basement membrane or the stromal matrix [28]. Basement membrane is one of the most important barriers against cancer cell invasion [28]. Therefore, for this study, we used BD Matrigel, a solubilized basement membrane preparation, isolated from the Engelbreth-Holm-Swarm mouse sarcoma, to model mimic gastric carcinoma invasion *in vivo*. Using a transwell chamber, we isolated the highly invasive subpopulation PAMC82-P3 from the parental PAMC82 cell line. *In vitro *selection provides a useful approach to isolate cell sub-lines with different invasion and metastatic potentials. Microarray analysis was used to determine the genes which may be involved in invasion, and *MMP28 *was one of the most interesting genes shown to be differentially regulated in PAMC82-P3 cells compared to PAMC cells.

MMP28 (epilysin), structurally belongs to the MMP19 subfamily, and represents one of the newest MMP member. MMP28 is expressed in normal and carcinoma tissues [29], basal keratinocytes at and surrounding wound edges [30], in fetal tissues and rhesus monkey placenta during early pregnancy [31], suggesting a role for MMP28 in normal tissue homeostasis, wound repair, and development, as well as in tumor progression [11,14]. MMP28 may also be involved in immune function, as it is expressed in normal circulating human T lymphocytes and is upregulated in osteoarthritic cartilage [32]. Few studies have investigated expression of MMP28 in human tumor samples; however, it is overexpressed in oral squamous cell carcinoma [11]. This study demonstrates MMP28 protein is overexpressed in gastric tumors (30/30, 100%) compared to normal epithelia (16/30, 53.3%, *p *< 0.001). MMP28 protein was expressed in gastric cancer cells and lymph node metastasis and not located in the surrounding normal tissues. This study also indicates MMP28 expression is significantly positively correlated with tumor invasion, lymph node metastasis and tumor-node-metastasis stage (*p *< 0.001), suggesting MMP28 plays a role in gastric carcinoma invasion and metastasis. Taken together, these data indicate MMP28 plays an important role in gastric cancer progression.

Illman SA et al. demonstrated expression of MMP28 altered cell phenotype towards a more adhesive, less migratory behavior [14]. However, biological evidence from *in vitro *and *in vivo *experiments has not yet clarified the relationship between *MMP28 *and cancer metastasis. In the current study we have shown, to our knowledge for the first time, that MMP28 positively regulates invasion of gastric cancer cells *in vitro *and can induce a metastatic phenotype *in vivo*. Increased expression of MMP28 led to a dose-dependent increase in invasive ability of N87 cells. These results provide the first evidence that MMP28 plays an important role in tumor invasion and metastasis and suggest MMP28 may be an effective target for suppression of metastasis in gastric cancer.

## Conclusions

We have established a gastric carcinoma invasion model using a highly invasive sub-line of tumor cells in which *MMP28 *was overexpressed. Further investigation revealed MMP28 is significantly correlated with invasive and metastatic ability and is a valuable marker of poor prognosis in gastric cancer. This study provides the first evidence that MMP28 can promote invasion and metastasis in gastric cancer.

## Competing interests

The authors declare that they have no competing interests.

## Authors' contributions

NJ designed and directed the study. PJ, TYF and ZZ finished the most of the experiments. WJ and ZXM coordinated data collection and quality control, and assisted in the interpretation of results. All authors read and approved the final manuscript.

## Pre-publication history

The pre-publication history for this paper can be accessed here:

http://www.biomedcentral.com/1471-2407/11/200/prepub

## Supplementary Material

Additional file 1 **Gene Array of PAMC82-P3 and PAMC82**. Click here for file

Additional file 2 **Q-RT-PCR analysis the expression of MMP28**. Click here for file
