# Supplementary material for: MMP28 (epilysin) as a novel promoter of invasion and metastasis in gastric cancer
Source: BMC Cancer. 2011 May 26;11:200. doi: 10.1186/1471-2407-11-200 (PMC3115915; doi:10.1186/1471-2407-11-200)
Supplement: Additional file 2 — Q-RT-PCR analysis the expression of MMP28. [file 1471-2407-11-200-S2.DOC]

**Expression of MMP28**

| Sample | U6Ct | MMP28Ct | MMP28Ct | MMP28Ct-U6Ct | 2 - **^△△^** ^CT^ |
| --- | --- | --- | --- | --- | --- |
|  |  |  | -U6Ct | - PAMC82 |  |
| PAMC82 | 10.45 | 24.85 | 14.4 | 0 | 1.00 |
| PAMC82 | 10.34 | 24.7 | 14.36 | -0.04 | 1.06 |
| PAMC82 | 10.26 | 24.17 | 13.91 | -0.49 | 1.44 |
| P1 | 11.26 | 24.54 | 13.28 | -1.12 | 2.22 |
| P1 | 11.11 | 24.66 | 13.55 | -0.85 | 1.83 |
| P1 | 11.26 | 24.8 | 13.54 | -0.86 | 1.83 |
| P2 | 11.27 | 23.36 | 12.09 | -2.31 | 5.06 |
| P2 | 11.27 | 23.44 | 12.17 | -2.23 | 4.78 |
| P2 | 11.11 | 23.22 | 12.11 | -2.29 | 5.00 |
| P3 | 11.52 | 22.91 | 11.39 | -3.01 | 8.22 |
| P3 | 11.45 | 23.09 | 11.64 | -2.76 | 6.94 |
| P3 | 11.36 | 23.3 | 11.94 | -2.46 | 5.61 |
